# P-1366. Outcome of 10 Patients with Colistin-Resistant Gram-negative isolates in a Tertiary Care Hospital in South India

**DOI:** 10.1093/ofid/ofae631.1543

**Published:** 2025-01-29

**Authors:** S Shibi, S Shobana, R Sanjai

**Affiliations:** Apollo Hospital, Chennai, Tamil Nadu, India; Apollo Hospital, Chennai, Tamil Nadu, India; Apollo Hospital, Chennai, Tamil Nadu, India

## Abstract

**Background:**

The emergence of colistin-resistant gram-negative isolates poses a significant challenge in the management of infections, particularly in tertiary care settings. This study aims to investigate the clinical outcomes of 10 patients with colistin-resistant gram-negative isolates in a South Indian tertiary care hospital. Understanding the impact of these resistant strains on patient outcomes is crucial for guiding treatment strategies and improving patient care in the face of increasing antimicrobial resistance.

**Methods:**

This study conducted between January 2023-April 2024 at a tertiary hospital in India. We reviewed patients’ clinical profile, outcome, and antibiotics that were used for treatment.

**Results:**

Ten colistin-resistant isolates were reported over 16 months. Respiratory was the most common source of the isolate, followed by blood and urine samples. Klebsiella pneumoniae constituted 50%, Acinetobacter baumannii 30%, Pseudomonas aeruginosa 20% of all isolates. Susceptibility of these isolates to other drugs were Amikacin 70%, tigecycline 58%, and chloramphenicol 25%. Antibiotics that were used for treatment combinations among the following antimicrobials as shown in Table 1.

**Table 1:** Patient profile, antibiotic combination used, and outcome

S.NO	SAMPLE	ANTIBIOTICS USED FOR TREATMENT	OUTCOME
1	Respiratory	Colistin +Tigecycline + Chloramphenicol	Death
2	Respiratory	Meropenem + Colistin	Death
3	Respiratory	Co-trimoxazole + Tigecycline	Discharge
4	Blood	Tigecycline + Chloramphenicol	Death
5	Pus	Amikacin + Colistin	Discharge
6	Urine	Chloramphenicol + Imipenem	Discharge
7	Respiratory	Colistin + Tigecycline	Death
8	Respiratory	Amikacin + Colistin	Discharge
9	Wound Swab	Fosfomycin + Colistin	Death
10	Respiratory	Meropenem + Colistin	Death

**Conclusion:**

Colistin resistance is becoming more common in Gram-negative bacteria, particularly K. pneumoniae, in Indian hospitals. Among patients infected with colistin-resistant gram-negative isolates, the mortality rate is 60%, while 40% are discharged. Amikacin, tigecycline, and chloramphenicol could be considered for combination therapy.

**Disclosures:**

**All Authors**: No reported disclosures

